# Defence mitigation by predators of chemically defended prey integrated over the predation sequence and across biological levels with a focus on cardiotonic steroids

**DOI:** 10.1098/rsos.220363

**Published:** 2022-09-07

**Authors:** Shabnam Mohammadi, Lu Yang, Matthew Bulbert, Hannah M. Rowland

**Affiliations:** ^1^ School of Biological Sciences, University of Nebraska, Lincoln, NE, USA; ^2^ Institut für Zell- und Systembiologie der Tiere, Universität Hamburg, Hamburg, Germany; ^3^ Wellcome Sanger Institute, Cambridge, UK; ^4^ Department of Biological Sciences, Macquarie University North Ryde, New South Wales, Australia; ^5^ Department of Biological and Medical Sciences, Faculty of Health and Life Sciences, University of Oxford Brookes, Oxford, UK; ^6^ Max Planck Institute for Chemical Ecology, Jena, Germany

**Keywords:** cardiotonic streroids, cardenolides, bufadenolides, arms race, predation

## Abstract

Predator–prey interactions have long served as models for the investigation of adaptation and fitness in natural environments. Anti-predator defences such as mimicry and camouflage provide some of the best examples of evolution. Predators, in turn, have evolved sensory systems, cognitive abilities and physiological resistance to prey defences. In contrast to prey defences which have been reviewed extensively, the evolution of predator counter-strategies has received less attention. To gain a comprehensive view of how prey defences can influence the evolution of predator counter-strategies, it is essential to investigate how and when selection can operate. In this review we evaluate how predators overcome prey defences during (i) encounter, (ii) detection, (iii) identification, (iv) approach, (v) subjugation, and (vi) consumption. We focus on prey that are protected by cardiotonic steroids (CTS)—defensive compounds that are found in a wide range of taxa, and that have a specific physiological target. In this system, coevolution is well characterized between specialist insect herbivores and their host plants but evidence for coevolution between CTS-defended prey and their predators has received less attention. Using the predation sequence framework, we organize 574 studies reporting predators overcoming CTS defences, integrate these counter-strategies across biological levels of organization, and discuss the costs and benefits of attacking CTS-defended prey. We show that distinct lineages of predators have evolved dissecting behaviour, changes in perception of risk and of taste perception, and target-site insensitivity. We draw attention to biochemical, hormonal and microbiological strategies that have yet to be investigated as predator counter-adaptations to CTS defences. We show that the predation sequence framework will be useful for organizing future studies of chemically mediated systems and coevolution.

## 1. Introduction

Predator–prey relationships belong to the most important and well-studied ecological interactions in nature. Prey evolve defences in response to selection from predators, which can be categorized according to the phase of the predation sequence in which they operate [1]. Prey can reduce the chance of *encounter* by avoiding habitats where predators are more common, the chance of *detection* through lack of movement and cryptic appearance [2,3], the risk of *identification* through mimicry or masquerade [4,5], and the likelihood of being *subjugated* and *consumed* with physical and chemical defences [5,6]. Predators, in turn, develop diverse sensory systems, speed, strength, learning and so on [1]. The interactions between predators and prey have often been regarded as an arms race or an example of coevolution but, in most cases, there is little evidence of coevolutionary responses by predators.

In this review, we use the predation sequence as a conceptual framework with the aim to understand the types of predator strategies that evolve in response to attacking chemically defended prey. This approach has been used successfully for many forms of prey defence and has led to significant insights into the evolution of these adaptations [5,7,8]. This method is particularly useful for predator mitigation strategies as it allows us to bring together a broad range of literatures to form a coherent research field that is better aligned with the broader predator–prey literature. Placing predator strategies into these categories also allows us to investigate whether generalized predation methods, which we define as methods that apply to many different types of prey, are more often found in the early stages of the predation sequence, and specialized methods—those that are more prey-specific—in the later stages. This was predicted by Endler over 30 years ago [1].

Just as those before us, who also attempted to bring together the literature on the evolution of predators in response to chemically defended [9], we focus our review on a specific interaction between predators and prey: in our case, those that involve cardiotonic steroids (CTS) as a chemical defence. In this system, coevolution is well characterized between specialist insect herbivores and their host plants [10], but evidence for coevolution between prey and their predators has received less attention. This system is especially compelling because of the widespread use of CTS as a form of chemical defence across the plant and animal kingdoms, which provides a rich body of comparative data. We start by introducing CTS and their history of research in predator–prey interactions, then briefly review the different prey animals that are defended by CTS and the predators that feed on them, before delving into the different methods that predators use to mitigate CTS defences across the predation sequence. We integrate these methods across biological levels of organization, from biochemistry, to physiology, to microbiology and to behaviour. We discuss the costs and benefits of attacking CTS-defended prey because this is integral for our understanding of the fitness consequences and selective pressure on predators and the ecological dynamics of predator–prey interactions. Our aim is to promote research that encompasses more integrative investigations of the diverse and multi-faceted mechanisms influencing the evolution of this system, and to suggest where researchers can focus their studies to shed light on whether a coevolutionary arms race is ongoing between predators and prey.

## 2. A brief introduction to and history of cardiotonic steroids in predator–prey evolution

**Figure 1.**
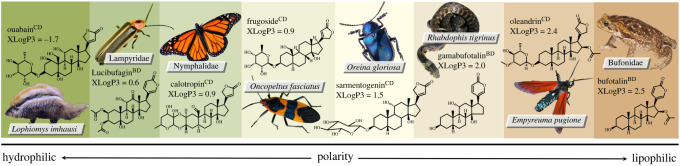
Axis of polarity of CTS produced or sequestered by animals. CTS polarity is represented by octanol-water partition coefficients (predicted by XLogP3). This is not an exhaustive list of CTS found in each prey source, but illustrates key characteristic compounds. Cardenolides (denoted by ^CD^), which are generally glycosylated, tend to have higher polarities than bufadienolides (denoted by ^BD^), which are not glycosylated. Polarity data were obtained from the National Center for Biotechnology Information's PubChem. Photo credits: crested rat (*Lophiomys imhausi*) by Don McCulley (2018); firefly (*Photinus* sp*.*) by Katja Schulz (2018); monarch butterfly (*Danaus plexippus*) by Peter Miller (2014); milkweed bug (*Oncopeltus fasciatus*) by Judy Gallagher (2017); cobalt milkweed beetle (*Chrysochus cobaltinus*) by Oregon Department of Agriculture (2016); tiger keelback snake (*Rhabdophis tigrinus*) by Yasunori Koid (2009); spotted oleander wasp moth (*Empyreuma affinis*) by Shaina Noggle (2010); cane toad (*Rhinella marina*) by Brian Gratwicke (2012). Information on sequestration of *O. fasciuatus* from Paola Rubiano Buitrago (pers. comm.).

CTS are a diverse group of compounds derived from triterpenoids that are found primarily in plants, but also in animals (figure 1; [10]) and have a specific physiological target, the transmembrane protein Na^+^, K^+^-ATPase (NKA, [11,12]). CTS are found in prey organisms on every continent, and their diversity and concentration are variable among prey species and individuals [13]. There are two classes of CTS: cardenolides and bufadienolides. Both are produced de novo in plants and animals [13], and some animals also sequester CTS from their host plants or prey [14,15]. This sequestration has almost certainly evolved as a defence against predators [16–19]. CTS are toxic because they bind to the extracellular surface of the transmembrane protein NKA [11,12] and, when bound, disable passage of Na^+^ and K^+^ across the membrane. This disrupts electrochemical gradients causing many physiological systems to become dysregulated [20]. Although the NKA is highly conserved among animals, independent evolution of NKA insensitivity to cardenolides has occurred in six taxonomic orders of insects that specialize on cardenolide containing plants [21].

In many cases, CTS consumption results in predators rejecting prey and learning to avoid them [16], which, in 40+ years of research, was decoded by Brower and co-workers [14,17,22,23]. Focusing on the monarch butterfly (*Danaus plexippus*) that as caterpillars feed on milkweed plants (*Asclepias*) and sequester cardenolides [10], Brower and co-workers revealed the chemical and pharmacological basis of the butterfly's chemical defence [24,25]. When *Asclepias*-fed monarchs were presented to blue jays (*Cyanocitta cristatata*) the birds consumed them and universally responded by vomiting, and subsequently avoided attacking the monarchs in future encounters [17]. Brower and co-workers also pioneered research on resistant predators, providing the first evidence for species of birds and rodents that were immune to the toxic effects of CTS [17,26–28]. They were the first to hypothesize that resistant predators had probably undergone changes to their gustatory systems, and that physiological resistance evolved in the ancestors of bird and rodent predators of monarchs—topics that we cover in §§5.2 and 6.2, respectively [29,30]. But, 30 years on, the role of NKA in CTS resistance of these bird and rodent predators have not been functionally studied, although predicted resistance-conferring genetic substitutions have been identified [31].

## 3. Taxonomic distribution and diversity of cardiotonic steroids in prey

The two main classes of CTS compounds—cardenolides and bufadienolides—differ in the structure of the steroid backbone and lactone group (the aglycone; figure 1). Cardenolides are primarily produced in plants and comprise a steroid backbone structure with a five-membered lactone group and a sugar moiety attached to C-3 of the first carbon ring [10]. The subset of CTS that possess a sugar moiety on C-3 are known as cardiac glycosides because their side chains are derived from sugars (are glycosylated). Bufadienolides have a six-membered lactone ring at C-17 and typically lack a sugar moiety [10]. Despite its frequent use in the literature, the term ‘cardiac glycoside' does not cover the majority of bufadienolides found in animals, which are non-glycosylated. For this reason, we use the umbrella term cardiotonic steroid, except for the cases where we can refer to specific CTS class.

Sequestration of dietary cardenolides is known from members of several Lepidoptera families, including Danaidae [32] and Arctiidae [33–36]. The sequestered cardenolide profile in monarch butterflies is dependent on host plant characteristics and larval developmental stage [23,37,38]. Several beetles synthesize their own cardenolides [39,40], and cardenolides have also been detected in Eurasian toads, *Bufotes viridis* [41] and African crested rats, *Lophiomys imhausi* [42]. Bufadienolides are most often found in toads (family Bufonidae) (reviewed in [43]), and the bufadienolide profiles from skin secretions of toads vary significantly from species to species, and even within species by population [44–49]. While CTS are found in the parotoid glands of adults, they have also been detected at lower concentrations in ovaries, oocytes, eggs, tadpoles, plasma and bile [43]. Lucibufagins—a subclass of bufadienolides—are believed to be synthesized from cholesterol by fireflies (mainly from the subfamily Lampyrinae). Species of the genus *Photuris*, which are members of the sister group to Lampyrinae, cannot synthesize their own lucibufagins and instead acquire them by preying on lucibufagin-producing fireflies [50–53]. Lucibufagins are also sequestered by keelback snakes of the genus *Rhabdophis* in a remarkable example of a dietary shift from eating toads to eating fireflies [54]. Other animals that are chemically defended by CTS include a wide range of insects that mostly sequester cardenolides from their plant hosts. Sequestering insects include beetles of the cerambycid genus *Tetraopes* and chrysomelid genus *Chrysochus* [55–57]; as well as some aphids (Homoptera: Aphididae, oleander aphid, *Aphis nerii* [58]), bugs (Heteroptera: Lygaeidae (*Oncopeltus fasciatus* and *Lygaeus kalmi* [55,59]) and grasshoppers (Orthoptera: Pyrgomorphidae [60]). Finally, several beetles are known to synthesize their own cardenolides. These include the chrysomelids of the genera *Oreina* [39] and *Chrysolina* [40], which use bright and conspicuous coloration to signal their chemical defences to predators, otherwise known as aposematism [5,61].

## 4. Taxonomic distribution of predators of cardiotonic steroids-defended prey

There are two main ways of classifying predators. One is to use a ‘trophic classification' method: carnivores consume animals, herbivores consume plants, and omnivores consume prey from more than one trophic level. Our preferred alternative is a ‘functional' classification of the type outlined in [62]: true predators, grazers, parasitoids and parasites. Using this definition, we searched Google Scholar for published records of predators feeding on CTS-defended prey. We also searched the natural history notes from herpetological reviews. We used the search strings that included the prey animals known to contain CTS defences and the types of CTS from section two with the word predator or predation. Our search strings included: Danaidae, Arctiidae, *Danaus*, monarch butterfly, Eurasian toad, toad, *Bufo*, Bufonidae, cane toad, *Bufotes viridis,* African crested rat, *Lophiomys imhausi*, fireflies, Lampyrinae, *Photuris*, snake, keelback snake, *Rhabdophis* cerambycid, *Tetraopes*, chrysomelid, *Chrysochus, Chrysolina Oreina* Homoptera, Aphididae, oleander aphid, *Aphis nerii,* Lygaeidae, milkweed bug, *Oncopeltus fasciatusi, Lygaeus kalmi*, grasshoppers, Orthoptera and Pyrgomorphidae. As taxonomic designations have changed repeatedly, especially among bufonidae ‘true toads', it was also necessary to work backwards and forwards from review articles and field guides which had citations using previous versions of species names. Only the current species names, reconciled from the Global Biodiversity Information Facility (GBIF), were used for the final list.

**Figure 2.**
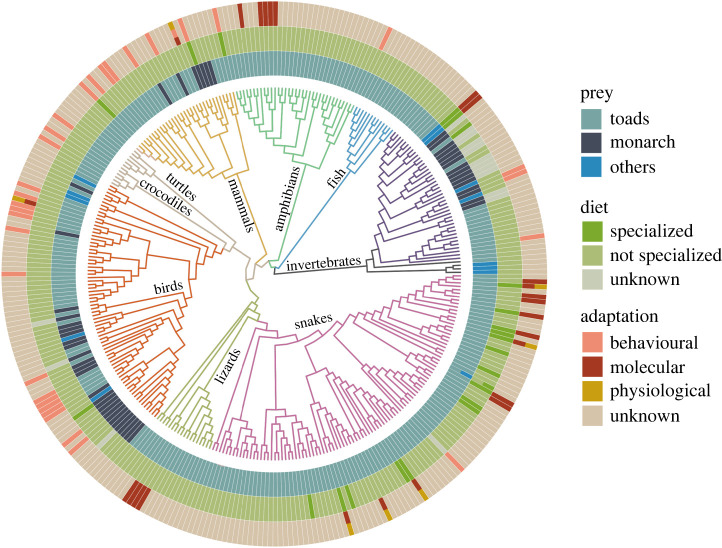
Phylogenetic tree of predators of CTS-defended animals including true toads (Bufonidae spp.) and milkweed butterflies (*Danaus* spp*.*), including monarchs. Information on behavioural, molecular and physiological adaptation is scarce and unevenly reported for different animal groups. Only those confirmed by functional experiments are marked as having molecular resistance to CTS. Phylogenetic relationships were inferred from timetree.org. References for prey, diet and adaptation characterizations are available in electronic supplementary material, table S1. Figure made with the phytools package in R.

After reviewing the titles and abstracts of the search results, we had a final dataset of 574 records of predation of CTS-defended prey (electronic supplementary material, table S1). These records include field observations as well as feeding studies with captive animals. Seventy three per cent of the reports related to the predation of toads, while the rest documented predators that feed on non-toad CTS-defended prey (lepidoptera, fireflies, grasshoppers, true bugs, beetles and aphids). Both anurans and caudates consume toads of one or more life stage, and toad eating is widespread among snakes (see [63] for a review). Entire genera either feed exclusively on toads or make toads a crucial part of their diet (e.g. hognose snakes (*Heterodon* spp*.* [64–68]), keelbacks (*Rhabdophis* spp*.*) [54,69,70]; night adders (*Causus* spp*.*) [71,72]; garter snakes (*Thamnophis* spp.) [73–75] and South American hognose snakes (*Xenodon* spp.) [76,77]). Toad eating is also observed in mammals (mustelids and rodents [78–80]), some shorebirds, waterbirds and waterfowl, and aquatic invertebrates that typically feed on eggs, hatchlings and tadpoles [81] (figure 2). One of the most remarkable predators of toads is the nymph of some epomis beetles [82–84], which captures juvenile toads with an elaborate luring strategy (see §5.1 on encounter [85]).

Over 30 vertebrate species are known to eat monarch butterflies with minimal adverse effects (electronic supplementary material, table S1); arthropod predators include lacewings, ants, spiders, ladybirds, cockroaches, mantids, predatory stink bugs, assassin bugs and wasps [86–91]. The most striking example of bird predators that have succeeded in breaking through the cardenolide defence of the monarch is the mixed- and single-species flocks of birds including the black-headed grosbeak (*Pheucticus melanocephulus*) and the black-headed oriole (*Oriolus larvatus*), which kill an average of 15 000 butterflies per day in the large overwintering aggregations in Mexico [26,92]. Species of mice that are found near monarch overwintering aggregations (including *Peromyscus aztecus*, *Reithrodontomys sumichrasti*, *Neotomodon alstoni* and *Microtus mexicanus*) also feed heavily on the butterflies. An individual deer mouse (*Peromyscus melanotis*) can consume an average of 37 monarchs each night [27]. Over the winter season, the mice account for approximately 5% of the total predation on the monarch colony (a population of *P. melanotis* can attack 100–3000 monarchs per night [27]. Paper wasps can also kill and eat substantial numbers of monarch larvae, in both controlled indoor experiments and in the wild where they fly from their nests to forage on plants containing larvae (up to 5000 monarch caterpillar larvae were killed and eaten over the course of one study by Rayor [93]). The paper wasp's choice of monarchs also varies depending on the species of milkweed on which the larvae have fed [93].

A number of vertebrates are known to eat the other main CTS-defended insects—fireflies [94]. Bats have been observed chasing firefly adults, but surprisingly only big brown bats (*Eptesicus fuscus* subsp. *fuscus*) have been confirmed to have fireflies in their diet [95]. Likewise, anoles such as *Anolis evermanni* and *A. cristatellus* have been suggested to be avid consumers of fireflies, while the likelihood of other anoles eating fireflies depends on their level of satiation [96]. The worm-eating clade of keelback snakes, which includes *Rhabdophis nuchalis* and *R. leonardi*, have shifted their regular diet of earthworms to occasionally include firefly larvae [54]. Doing so allows them to sequester lucibufagins from the fireflies for use in their own chemical defence (see §5.1).

We classified predators as generalist if they tend to exploit a wide variety of resources, although they might exploit one if it is very abundant, and specialists if they are species adapted to exploit a single food type or niche, but will exploit other niches either opportunistically or when primary food is in short supply [97]. From this we found that generalist predators made up 84% of our records while 6% could be considered as specialists (including birds, insects, mammals and reptiles). We found that behavioural adaptations were more often reported in generalists than specialists, and that molecular resistance (confirmed by functional assay) is present in both generalists and specialists, but has been tested in only 5% of the predators known to eat CTS-defended prey. For the majority of specialists, whether their degree of CTS tolerance matches prey-specific defensive chemistry remains untested.

## 5. How do predators overcome cardiotonic steroids defences?

In the following sections, we uncover the potential evolutionary relationships between CTS-specific defences and predator adaptations at the different stages of predation. Our intention is not to provide an exhaustive list of all mitigation strategies, but to provide the reader with an idea of the diversity and parallelism of these strategies, and a simple way in which they can be categorized.

### 5.1. Encounter

The first stage of predation is for predators to situate themselves such that they increase their chances of encountering CTS-defended prey. Prey abundances and distributions change over time and space, which creates a complex changeable environment [98]. The life history and demographics of different predators can increase the probability of their encountering CTS-defended prey. For example, the common frogs (*Rana temporaria*) breed earlier and their offspring develop faster than natterjack toads (*Epidalea calamita*)*,* which allows common frog tadpoles to eat toad spawn and newly hatched tadpoles, and can result in 100% toad mortality [99]. Predators also move to areas where CTS prey are found. Adult *P. melanotis* migrate in large numbers to areas where monarch butterflies aggregate in the winter and feed on monarchs and breed successfully, whereas four other species of mice do not breed because they are deterred by the monarchs' defences [30]. Predators can also lure prey during encounters, as seen in trophic role reversal by larvae of ground beetles (genus *Epomis*; [85]). Larvae of *E. circumscriptus* and *E. dejeani* move their antennae and mandibles in the presence of frogs and toads, which triggers amphibian predation behaviour. The larvae avoid the predator's attack by ignoring toe wagging by the amphibians, and instead attach to the amphibian's body and start feeding.

### 5.2. Detection, identification and approach

After encountering potential prey, predators must detect and decide whether the prey are worth attacking. Deciding to approach CTS-defended prey requires a predator to overcome the initial reluctance that most naive individuals express after encountering CTS-defended prey [100,101]. This can be facilitated and maintained via intergenerational cultural transfer [102], i.e. foraging by older individuals who consume chemically defended prey without ill effects can locally enhance foraging by younger less experienced predators (i.e. optimal action is to shift to attacking the prey [103,104]). Social transmission of prey approach and handling has been suggested for black-headed grosbeaks (*Pheucticus melanocephalus*) that feed on monarch butterflies [17], and by Torresian crows (*Corvus orru*) that feed exclusively on the non-toxic parts of toads [105]. Socially acquired prey preferences can also be modified later in life [106]. For example, fringe-lipped bats (*Trachops cirrhosis*) acquire a novel association between the call of a toad species and palatable prey after observing the positive foraging experience of a conspecific [107]. This type of reversal learning, wherein predators adjust their prey choices based on stimuli, is important when thinking about the identification and fitness reward of auto-mimics (e.g. monarch butterflies that lack cardenolides). If predators could enhance their identification of prey profitability through social transmission, then they could influence how frequency-dependent selection operates on prey [104]. Because social transmission of avoidance is beneficial for defended prey [104] we would expect selection to favour prey to evolve traits that maximize opportunities for social learning about identification such as new, perhaps more salient, multi-modal defences [108] that increase distastefulness to elicit strong disgust responses [109]. The three systems (grosbeaks, crows and bats) present compelling opportunities to test the role of social information of different populations of predators' attack decisions (identification stage) and capture (approach stage) and the potential for reciprocal responses by prey.

### 5.3. Subjugation

Once predators have approached prey they must handle and subdue them. We found that dissecting behaviour is a common trait in predators (figure 2), including insects [110–112], mammals and birds [105,113,114], and even in limbless predators such as snakes [115]. At first glance, dissecting behaviour is a surprising evolutionary solution for snakes. However, it is made possible because of the enlarged posterior maxillary teeth [115] which are thought to have evolved to allow deep tooth penetration into prey, as well as for other non-predatory purposes such as male–male combat [116]. Dissecting behaviour is innate in some mustelids [78,117,118], and in some birds this behaviour is thought to be exapted from fruit-eating, and would therefore be of low cost to maintain given its benefit in other contexts [17,92]. Dissecting behaviour may evolve and be maintained via cultural transfer [105] because headshaking in response to aversive stimuli could be used by conspecifics to guide dissecting behaviour [119], and for individuals to develop discriminatory chemosensory behaviour [17].

The widespread occurrence of dissecting behaviour suggests a shared ability to taste and avoid CTS in predators [120]. Although cardenolides are often described as bitter tasting compounds [121], we lack specifically designed comparative tests on the chemosensory detectability of CTS. Japanese tiger keelback snakes (*Rhabdophis tigrinus*) show no discrimination between purified bufadienolides and control stimuli [122], which suggests that there are other chemosensory signals that the snakes use during predation. On the other hand, single cardenolides do elicit taste discrimination by birds and this varies with cardenolide polarity [17]. In adult monarch butterflies, cardenolides are nearly twice as concentrated in the wings than the rest of the body and are especially concentrated in the wing-scales, which gives predators that attack this part of the body a mouthful of bitter compound [34]. Whether this is an evolutionary response to predation, and whether predators that attack monarchs vary in their ability to detect and tolerate cardenolides in a manner that matches the concentration in the wings is yet to be systematically investigated but could be evidence of differential coevolution.

Some predators, such as *P. melanotis*, and European hedgehogs (*Erinaceus europaeus*), which feed on CTS-defended prey, have significantly higher taste rejection thresholds for single cardenolides, monarch butterflies and cardenolide-defended grasshoppers (*Poekilocerus bufonius*) compared with other closely related species that do not feed on CTS-defended prey [30]. This taste insensitivity may be an adaptation to let predators consume CTS-defended prey, and there appears to be sufficient intraspecific variability in this behaviour to have resulted from natural selection, but this is yet to be investigated [28]. Taste insensitivity to cardenolides suggests that either the taste receptor genes have undergone functional changes, or that the valence of CTS has changed, or can be changed, from negative to positive. Future research comparing the comparative responses of predators combined with comparisons of the g-protein-coupled Tas2r taste receptors responsible for bitter taste perception could reveal patterns of evolution related to prey defences and predator diet, and test whether taste insensitivity is paired with a detoxifying metabolism or target-site insensitivity (TSI) [123,124].

### 5.4. Consumption

Evolved avoidance of CTS by dissecting or eating the least CTS-laden parts of prey is one possible result of predator–prey interactions. In this section, we describe TSI via amino acid substitutions in the CTS binding pocket of the NKA and its potential as a candidate for predator–prey coevolution. If an arms race-type process is occurring, we expect matched levels of CTS defence of prey and resistance ability of the predator [9].

Most vertebrates possess three paralogs of the NKA subunit α gene (*ATP1A1-3*) that have tissue-specific expression profiles and are associated with distinct physiological roles. Most amino acid variation among species and paralogs is concentrated in the first extracellular loop (residues 111–122; H1–H2 loop), which makes up part of the CTS binding domain and shows clade- and paralog-specific patterns of variability but also shows remarkable patterns of convergence, parallelism and divergence [125]. Amino acid substitutions at sites 111 and 122 in particular have been found to be key in the evolution of TSI in insect and vertebrate species [21] and have evolved in snakes [63,126], frogs [127,128] and other vertebrates [125].

Many birds that are sympatric with invasive toads, but have no evolutionary history of co-occurring with toads, have no amino acid substitutions likely to confer resistance [129]. Snakes that have shifted their diet from eating toads to eating fireflies do have TSI [54]. It has been hypothesized that the black-headed grosbeak which feeds on monarch butterflies also possesses amino acid substitutions in two of the three paralogs which likely confer resistance [31,130]. In other species of birds that are reported as specialist feeders of CTS-defended danaid butterflies [131], such as bulbuls (*Pycnonotus barbatus*) and hornbills (*Lophoceros eucomelas*), genome annotations of *ATP1A1* of related species also show potential TSI-conferring substitutions in both *ATP1A1* and -*A2*. In other predators, such as the generalist egg parasitoid wasp, *Trichogramma pretiosum*, and in the generalist entomopathogenic nematode *Steinernema carpocapsae*, potential TSI-subsitutions are also present [31].

Understanding the evolutionary history and potential for coevolution of a trait requires some knowledge of the patterns of variation among individuals, populations and species [132]. Where functional tests of TSI substitutions have been performed, there can be greater than 10-fold variation in TSI among enzymes that have identical paired states at 111 and 122 [125], as well as significant variation in enzyme activity, which together suggest that substitutions at other sites also contribute to CTS resistance through intramolecular epistasis and can be subject to selection [31,133]. Enzyme function, however, is but a proxy for predicting effects on organismal fitness, and research exploring how the effects of adaptive mutations at the protein level cascade to the whole-organism fitness, and how they match the defences of prey in different populations and locations will be necessary to understand the potential for coevolution.

## 6. Mitigation strategies after consumption yet to be explored

**Figure 3.**
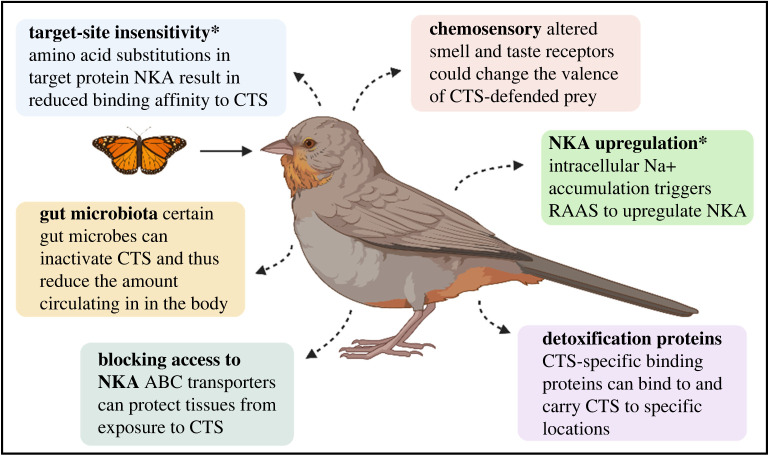
Summary of different potential mechanisms that can contribute to resistance in predators of CTS-defended prey. Mechanisms that have been empirically linked to contributing to a predator's ability to overcome CTS toxicity of defended prey are marked by an asterisk. Predators may avoid feeding on prey parts with high concentrations of CTS (e.g. [120]) or detoxify CTS after ingestion (e.g. [143]). In addition, they may possess altered target sites that are no longer susceptible to the toxic action of CTS [125]. Some predators sequester CTS from their prey and defend themselves against their own predators (e.g. snakes of the genus *Rhabdophis* [15]). Less attention has been paid to metabolic transformations that allow predators to detoxify CTS and excrete the resulting metabolites. These diverse mechanisms can influence a predator's behaviour, which in turn influences ecological interactions and ecological structures. Figure created with BioRender.com.

If CTS-consuming animals do not use TSI to avoid intoxication, how do they survive? Insect and vertebrate CTS can vary ontogenetically [23,37,38] from species to species and within populations [44–49], in terms of concentration, diversity [10] and polarity, which can influence their chemosensory detectability [17], toxicity [134], transport [135] and excretion [136]. CTS also vary seasonally and geographically [137,138], which may influence selection for TSI. In this section we draw on the information from plant–herbivore interactions where insects that possess sensitive NKA still feed on cardenolide-defended plants [36,139,140]. We discuss how predators could possess guts that are impermeable to cardenolides via biological barriers [141,142]; how hormonal systems can mitigate loss of NKA activity; and the scope for gut microbiota to neutralize the toxicity of CTS (see figure 3).

### 6.1. ATP-binding cassette transporters and binding proteins

One method to avoid CTS toxicity which has not been explored in predators is having an impermeable barrier to non-polar CTS [144,145]. Polar and hydrophilic CTS are unable to passively cross the gut and perineurium due to epithelial diffusion barriers such as septate junctions, and thus pass through predator bodies without causing toxicity [135]. But for non-polar CTS, the presence of P-glycoprotein efflux carriers, which are well known for their function in maintaining the blood–brain barrier of animals and have been identified in gut epithelial cells, could increase resistance to the toxins. Indeed, mice with P-glycoprotein deficiencies (mdr1a gene knockouts) respond with increased CTS levels in their tissues (particularly in the brain) after intravenous injections of the toxins compared with wild-type mice [146].

Binding proteins could also contribute to CTS resistance in predators [147]. Binding proteins typically transport non-polar steroid hormones through the bloodstream to their target cells, where in some cases interactions with docking proteins cause them to release the steroids [11,148]. Because endogenous CTS function in regulating cardiac contractility and circulation [20], it is possible that a binding protein system for transporting CTS to specific targets such as cardiomyocytes is already in place. Previous studies have shown that mammals possess a CTS-specific binding protein, which binds to the steroids with high affinity and inhibits their function [149,150]. These binding proteins are produced at high concentrations in the kidneys, where they probably protect the NKA of those tissues [148]. Gene sequences for these proteins, however, are still lacking and we do not know whether such a mechanism could provide substantial protection to a predator that ingests high concentrations of CTS.

### 6.2. Renin–angiotensin–aldosterone system and the enlargement of adrenal glands

**Figure 4.**
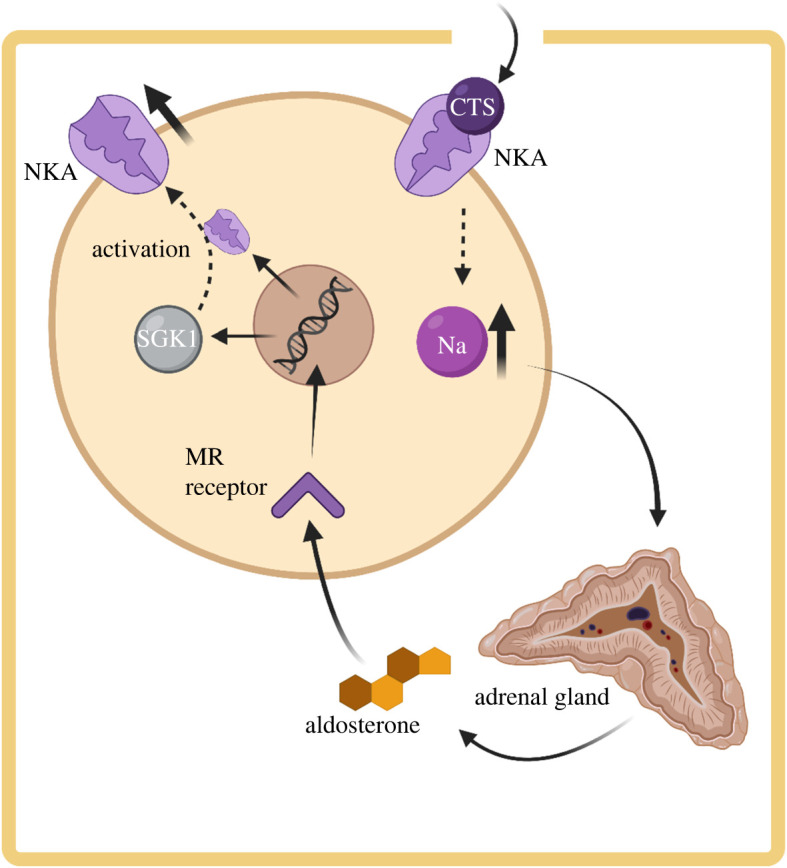
A schematic diagram of how the adrenal glands can signal the expression of NKAs following CTS exposure. CTS enters the organism, reaches a cell and disables NKAs, causing an increase in intracellular Na^+^ because the disabled proteins no longer transport Na^+^ out of the cell. This triggers the adrenal glands to secrete the mineralocorticoid (MR) hormone aldosterone, which passes through the cell membrane and binds to an intracellular MR receptor. This receptor translocates into the nucleus where it activates a transcriptional program inducing expression of modulators of sodium transport such as SGK1 and also NKAs themselves. Figure created with BioRender.com and based on data from [153,154].

A particularly interesting morphological pattern that has been identified in snakes that feed heavily on toads is extreme adrenal gland enlargement [151], which suggests that the renin–angiotensin–aldosterone system (RAAS) could play a role in mitigating CTS toxicity. Increased physiological stress from processing CTS could lead to higher production of stress hormones (i.e. corticosteroids and catecholamines) that results in adrenal enlargement. However, hormonal responses to bufadienolides in *Rhabdophus tigrinus* show no increase in circulating corticosteroid levels in response to bufadienolide injections [152]. Alternatively, increased production of the mineralocorticoid hormone aldosterone in the enlarged adrenal glands could compensate for reduction in NKA activity caused by CTS by increasing NKA expression (figure 4) [155,156]. Increased circulating aldosterone has been identified in the Japanese toad-eating snake *R. tigrinus* [152], which exhibits highly enlarged adrenal glands [151]. Furthermore, garter snakes (*Thamnophis elegans*) injected with bufadienolides responded with significantly increased NKA expression in their heart tissue [157]. However, whether CTS exposure directly leads to increased circulating aldosterone and NKA expression, and consequently adrenal gland enlargement in resistant predators, requires further experimental tests.

### 6.3. Gut microbiota

Gut microbiota are known to neutralize the toxicity of CTS by metabolizing CTS to reduced/inactivate compounds such as digoxin to dihydrodigoxin [143]. The bacterial source of digoxin metabolism has been traced to the Actinobacterium *Eggerthella lenta*, and the mechanism is linked to a multi-gene operon known as the cgr (cardiac glycoside reductase) [143,158]. In the presence of digoxin cgr genes are significantly upregulated, allowing *E. lenta* to inactivate digoxin by reducing its lactone ring (i.e. dihydrodigoxin). This modification is believed to distort the ring planarity leading to reduced binding to NKA. The cluster of genes that make up the cgr operon includes eight genes, which are present in individuals that can metabolize digoxin and are absent in non-metabolizers, thus representing a single genetic locus predictive of digoxin metabolism. Functional tests of one of the eight genes (*Cgr2*) show that it is sufficient for digoxin inactivation [159] and that it has strict specificity for cardenolides (e.g. digoxin, ouabain, ouabagenin, digoxigenin, digitoxin). How widespread gut bacteria that can digest CTS are in predators, and whether they are a key step to a predator's adaption to CTS-defended prey, remain open questions.

## 7. Benefits and costs of consuming cardiotonic steroids

Having covered the range of known and potential predator mitigation strategies we now discuss the costs and benefits of these strategies. This is necessary if we are to draw conclusions about the selective pressure on predators and therefore the ecology and evolution of these strategies. Many of the examples we have described in §§5 and 6 could be applied to any chemical defence mitigation, and likewise many of the benefits could also apply broadly. For example, coping with toxic prey can expand predator niches by providing a competitive release [160] as seems to be the case with the population of scansorial black-eared mice (*P. melanotis*) that are larger, heavier and reproduce more than mice of the same species whose territories are outside of the overwintering monarch roosts [27]. Thus, in this section, we cover the specific aspects of predator counter-adaptations to CTS, propose a range of putative benefits and costs, and suggest how these could be measured.

### 7.1. Defence against a predator's own enemies

Some predators sequester CTS from their diet for redeployment in their own chemical defence. Hedgehogs self-anoint skin secretions from toads onto their spines [161]; African crested rats (*Lophiomys imhausi*) have hairs that are highly specialized to wick up and store the cardenolide that they chew from the roots and bark of *Acokanthera schimperi* (Apocynaceae) [42]. When threatened during approach, these two very different species have evolved similar behaviours and warning displays: African crested rats part the hairs along their flank to reveal both warning coloration and their poison-laced hairs. Similarly, Japanese tiger keelback snakes (*Rhabdophis tigrinus*), which store bufadenolides in specialized nuchal glands on the back of their necks [15], arch their necks towards the threat revealing brightly coloured yellow and red skin covering the nuchal glands [162]. In some cases, pressure created by the arching of the neck breaks the skin, causing the stored toxins to shoot out towards the attacker (experienced personally by S.M.). Japanese tiger keelback snakes also maternally provision bufadenolides to their offspring via embryonic transfer. Gravid female snakes have been found to actively forage for toads [163], and the amount of CTS in the nuchal glands of offspring corresponds proportionally to the amount found in the mother [164]. The few members of *Rhabdophis* that have shifted their diets away from frogs and toads to smaller invertebrate prey occasionally feed on CTS-defended firefly larvae to maintain the defence benefit provided by CTS sequestration [54]. Whether other predators such as black-headed grosbeaks use cardenolides for defence without active sequestration mechanisms, as has been found for other organisms that tolerate toxin consumption [165], is an open question. This is possible, given that other species of grosbeak appear to have toxins in their feathers [166], and their orange and black colour could give them a transient defensive advantage against their own predators.

Many species of bufophagous (i.e. toad-eating) snakes also death feign in response to an attack [64,167–169]. The behaviour is not exclusive to bufophagous snakes, and at least one species of highly bufophagous snake (*Causus rhombeatus*) does not feign death [170]. However, the enlargement of the adrenal glands in several species of bufophagous snakes [151] is thought to be linked to this behaviour. Increased catecholamine production by enlarged adrenal glands could lead to a parasympathetic syndrome preceding death feigning [170]. Because CTS may render toad-eating snakes distasteful, ‘death feigning' may slow a predator's attack and increase the predator's detection of CTS [171].

Beyond chemical defence sequestration, predators may take advantage of CTS to protect themselves from parasites [172,173]. ‘Self-medication' [174] has not been investigated in predators that feed on CTS, but the diverse pharmacological properties of these compounds suggest that such an evolutionary relationship is possible. For example, several bufadienolides have been shown to have antimicrobial and antifungal properties [49,175]. Further, monarchs are well known to use cardenolides for self-medication, whereby they protect their eggs from parasites by laying them on CTS-defended milkweed plants [176].

### 7.2. Behavioural, physiological and molecular costs

As generally expected for adaptations, CTS resistance comes with a cost, but the evidence for this is scarce and indirect. Dissecting behaviour and slower prey handling may translate into an overall cost in fitness in some species [177]. Otters, for example, can ingest frogs immediately but require more time to skin, wash and select the parts to ingest from a toad [113]. Black-headed grosbeaks (*Pheuticus melanocephalus*) and orioles (*Icterus parisorum*) that feed on monarch roosts feed on a 7.85 day on-off cycle [102], and also change their feeding depending on ambient temperature [102], which is probably due to the changes in toxicity with ambient temperature [178,179]. Shifts in feeding patterns probably reduce the impact of cardenolide toxicity but increase opportunity costs of foraging over short windows of time. Whether this behaviour is evidence for detoxification costs or is a cost of TSI requires further study. In mice, introducing resistance-conferring substitutions that occur in wild-type *ATP1A1* onto *ATP1A2* negatively affects their learning ability, locomotor activity and anxiety-related behaviours [180]. A similar trade-off has also been observed in Australian snakes that feed on toads, which show reduced performance, locomotor capability and increased prey handling time compared with non-toad eaters [181,182].

Endowing a protein with a new function through mutation often incurs a cost, particularly with respect to the protein's original function [125,128,140,183]. Functional studies of TSI have repeatedly shown that resistance-conferring substitutions often carry substantial functional costs to the ATPase activity of NKA [125,128]. These negative pleiotropic effects can have major implications at higher biological levels due to the vital role that NKAs have in the maintenance of physiological homeostasis. Animals that have evolved TSI through substitutions at sites 111 and 122 have thus either co-adapted additional substitutions that compensate for such negative pleiotropic effect [128,140,183] or, as is the case with neotropical grass frogs of the genus *Leptodactylus* (Leptodactylidae) that feed on toads (but do not specialize on them), undergone a tandem duplication of *ATP1A1* and subsequent neofunctionalization of one copy, which allows them to maintain highly resistant and highly functional versions of the protein [128,133].

## 8. A broader view of cardiotonic steroids resistance in predators and the coevolution with cardiotonic steroids defences in prey

In this review, we have drawn together the evidence about the methods that predators use to overcome the suite of defences deployed by CTS-defended prey. We have shown that dissecting behaviour is used by invertebrates, reptiles, birds and mammals; that changes in perception of risk and of taste perception have occurred in mammals and birds; and that TSI via amino acid substitutions in the CTS binding pocket of the NKA has evolved in parallel in invertebrate and vertebrate predators. We have also pointed to biochemical, hormonal and microbiological strategies that have yet to be investigated in this context.

Using the predation sequence as a framework [1], it becomes apparent that variation in the consequence of the interaction between a predator and prey influences the strength of selection on defence mitigation strategies by predators (see also [184–186]). This difference in the selective pressure between the early and late stages of predation supports a pattern predicted by Endler [1], which is a tendency for many generalized predation methods to have evolved in the early stages of predation (optimal foraging, social learning, dissecting behaviour, changes in gustatory perception). There are three potential reasons for this. (i) Generalized methods may be less expensive than specialized methods because they are used continuously and for other purposes such as finding mates and holding territories, or are evolutionary responses to the predator's own predators or competitors [187]. For example, detecting and identifying CTS-defended prey is based on general sensory and cognitive properties such as diverse sensory systems, learning ability and primarily fit within optimal foraging theory [188]. In many cases, predators choose prey on the basis of their overall availability and profitability [189,190]. (ii) Because prey defences that operate early in the sequence are generalized and only generalized methods are required to overcome them, but in the later stages of the sequence prey defences are more specific and the risk to predators increases, with predators ‘forced' into experiencing selection (also proposed by Brodie III and Brodie Jr. [9]). Finally, (iii) the interactions between predators and CTS-defended prey are diffuse due to the community complexity of these natural predator–prey systems [191]. Our analysis shows that most predators prey on several species, and therefore the total selective pressure on each other is more diffuse.

Understanding the evolutionary history and potential for coevolution of a trait requires some knowledge of the patterns of variation among individuals, populations and species. This is well known for CTS-defended prey, but is still generally lacking for predators. Our review has highlighted potential areas to explore in predators: chemosensory perception, TSI, toxin-binding proteins and gut microbiota. This research field will benefit from more detailed within- and between-population analyses of these traits to quantify individual variation, which is necessary for selection to act. In many cases, it appears that predators are pre-adapted to feeding on CTS, i.e. muroid rodent TSI. Reconstructions of the evolutionary history of predators and co-occurrence with CTS prey, and their dietary specialization on—or tolerance to—CTS-defended prey will be important for understanding whether these animals are pre-adapted to attack CTS-defended prey [29,192] or whether TSI evolved directly from exposure to CTS, and whether there is evidence for ongoing coevolution.

## 9. Conclusion and future directions

Understanding the full range of mechanisms contributing to toxin resistance in predators of toxic prey is an important goal for evolutionary biology. The recurring emergence of predators that can feed on and exploit CTS-defended prey has involved remarkable convergence in the behaviours, physiology and molecular mechanisms by which they achieve this adaptation. Although a majority of research focus has revolved around TSI of NKAs, we have found that there are multiple physiological, chemosensory, behavioural and ecological mechanisms that can also contribute to, and consequently shape, the ability to overcome the CTS defence of prey. In table 1, we list key questions that could be addressed in our continued quest to understand the mechanisms that have shaped this adaptation.

**Table 1.** List of open questions for future studies aiming to expand our understanding of the mechanisms of CTS resistance in predators of toxic prey.

question	experimental scheme(s) to address question
chemosensory	
How do the taste receptor genes of CTS-resistant predators compare with those of sensitive predators?	Comparing the Tas2r genes of *Peromyscus* species that have varying sensitivity to cardenolides compared with related species of mice would reveal the underlying molecular mechanisms of CTS tolerance.
Are predators that dissect able to chemically identify CTS-laden tissue?	Modifying either real or artificial CTS-defended prey so that the CTS are stored in different parts of the body and observing the dissecting behaviour of predators would reveal whether they consistently avoid the same part of the body or whether they can detect CTS and avoid whichever part of the body contains it.
molecular mechanisms of resistance	
Are ABC transporters protecting additional tissues in predators of CTS-defended prey?	P-glycoprotein transmembrane proteins are encoded by the ABC (ATP-binding cassette) transporter gene superfamily [193]. The genes encoding these proteins fall into seven subfamilies (A–G) and have ancient eukaryotic origins [194]. ABCG2 or ABCG2-like genes have been found in 41 bird species, and ABCG2-like genes have been lost in only five species [195]. We recommend sequencing the ABC transporters and comparing expression patterns in resistant and non-resistant predators to determine whether these proteins are upregulated to protect important tissues. It is possible to express ABC transporters in cell culture to assay their ability to bind to relevant CTS [196] and such studies would confirm their ability to protect tissues. Exploring the coevolution of ABC and *ATP1A* genes in predators will be a key step in understanding the stages of evolution of CTS resistance.
Are binding proteins helping to protect tissues from CTS?	Isolating binding proteins from plasma and sequencing amino acids would help identify the gene(s) encoding these proteins.
	Measuring plasma levels of these binding proteins in resistant versus non-resistant predators would reveal whether they play an adaptive role in predators of CTS-defended prey.
physiological mechanisms of resistance	
Does the RAAS play a role in CTS resistance?	Rearing hatchling CTS-resistant animals (snakes or mice) on a diet with and without CTS and then monitoring circulating aldosterone levels on a long-term basis, followed by comparing adrenal gland morphology and tissue-specific NKA expression levels, would reveal if and how the RAAS system adapts to a CTS-heavy diet.
Are there physiological costs to resistance?	Investigating the effects of amino acid substitutions in *ATP1A* genes *in vitro* and *in vivo* with CRISPR-Cas9 would reveal how pleiotropic effects at the protein level cascade to the whole-organism level. This could subsequently reveal what physiological systems might be co-adapted with TSI.
Are there physiological costs to feeding on CTS?	Comparing the physiology and performance of CTS-resistant predators fed CTS-defended prey (toads) versus control prey (non-toad frogs) would reveal whether digesting the compounds is physiologically demanding and provide insights into the cost of this adaptation.
role of gut microbiota	
How widespread are gut bacteria that can digest CTS and are they key to a predator's adaption to CTS-defended prey?	Comparing CTS-metabolizing ability of stool cultures from predators of CTS-defended prey and those that avoid them would reveal whether there are CTS-metabolizing bacteria in the guts of predators.
	Comparing the composition of the microbiota between predators of CTS-defended prey and those that avoid such prey would reveal potential CTS-metabolizing strains.
	Inoculating germ-free resistant and non-resistant predators with CTS-metabolizing strains would reveal whether gut microbes can augment resistance or confer resistance on their own.
Are there *cgr* genes in the gut microbiome of cardenolide-feeding animals?	Because *cgr* genes were found to be responsible for the ability of some bacteria to metabolize cardenolides, a screen for these genes in the microbiomes of resistant and non-resistant species could point to whether gut microbiota contribute to CTS resistance in predators of CTS-defended prey.
behaviour	
Are some CTS-feeding animals self-medicating against parasites?	The Japanese tiger keelback snake (*Rhabdophis tigrinus*) is known to have high and highly variable parasite loads [197,198]. These snakes feed on toads and sequester bufadienolides into specialized nuchal glands on the back of their necks. The amount of bufadienolide in their nuchal glands directly correlates with the number of toads they have ingested. Measuring their bufadienolide contents and parasite loads would reveal whether they correlate with one another.

## Data Availability

Data used in this review are available in the electronic supplementary material, table [199].

## References

[1] Endler JA. 1991 Interactions between predator and prey. In Behavioural Ecology, 3rd edn (eds JR Krebs, NB Davies), pp. 169-196. Oxford, UK: Blackwell Scientific Publications.

[2] Merilaita S, Scott-Samuel NE, Cuthill IC. 2017 How camouflage works. Phil. Trans. R. Soc. B **372**, 20160341. (10.1098/rstb.2016.0341) 28533458 PMC5444062

[3] Stevens M, Ruxton GD. 2019 The key role of behaviour in animal camouflage. Biol. Rev. **94**, 116-134. (10.1111/brv.12438) 29927061 PMC6378595

[4] Skelhorn J, Rowland HM, Ruxton GD. 2010 The evolution and ecology of masquerade. Biol. J. Linn. Soc. **99**, 1-8. (10.1111/j.1095-8312.2009.01347.x)

[5] Ruxton GD, Allen WL, Sherratt TN, Speed MP. 2019 Avoiding attack: the evolutionary ecology of crypsis, aposematism, and mimicry. Oxford, UK: Oxford University Press.

[6] Blum M. 2012 Chemical defenses of arthropods. Amsterdam, The Netherlands: Elsevier.

[7] Bond AB. 2007 The evolution of color polymorphism: crypticity, searching images, and apostatic selection. Annu. Rev. Ecol. Evol. Syst. **38**, 489-514. (10.1146/annurev.ecolsys.38.091206.095728)

[8] Lindström L, Alatalo RV, Mappes J. 1997 Imperfect Batesian mimicry—the effects of the frequency and the distastefulness of the model. Proc. R. Soc. Lond. B **264**, 149-153. (10.1098/rspb.1997.0022)

[9] Brodie III ED, Brodie Jr ED. 1999 Predator-prey arms races: asymmetrical selection on predators and prey may be reduced when prey are dangerous. Bioscience **49**, 557-568. (10.2307/1313476)

[10] Agrawal AA, Petschenka G, Bingham RA, Weber MG, Rasmann S. 2012 Toxic cardenolides: chemical ecology and coevolution of specialized plant–herbivore interactions. New Phytol. **194**, 28-45. (10.1111/j.1469-8137.2011.04049.x) 22292897

[11] Schoner W. 2002 Endogenous cardiac glycosides, a new class of steroid hormones. Eur. J. Biochem. **269**, 2440-2448. (10.1046/j.1432-1033.2002.02911.x) 12027881

[12] Laursen M, Gregersen JL, Yatime L, Nissen P, Fedosova NU. 2015 Structures and characterization of digoxin- and bufalin-bound Na^+^, K^+^-ATPase compared with the ouabain-bound complex. Proc. Natl Acad. Sci. USA **112**, 1755-1760. (10.1073/pnas.1422997112) 25624492 PMC4330780

[13] Krenn L, Kopp B. 1998 Bufadienolides from animal and plant sources. Phytochemistry **48**, 1-29. (10.1016/s0031-9422(97)00426-3) 9621450

[14] Malcolm SB. 1994 Milkweeds, monarch butterflies and the ecological significance of cardenolides. Chemoecology **5**, 101-117. (10.1007/BF01240595)

[15] Hutchinson DA, Mori A, Savitzky AH, Burghardt GM, Wu X, Meinwald J, Schroeder FC. 2007 Dietary sequestration of defensive steroids in nuchal glands of the Asian snake *Rhabdophis tigrinus*. Proc. Natl Acad. Sci. USA **104**, 2265-2270. (10.1073/pnas.0610785104) 17284596 PMC1892995

[16] Brower LP, Ryerson WN, Coppinger LL, Glazier SC. 1968 Ecological chemistry and the palatability spectrum. Science **161**, 1349-1350. (10.1126/science.161.3848.1349) 17831347

[17] Brower LP, Fink LS. 1985 A natural toxic defense system: cardenolides in butterflies versus birds. Ann. N. Y. Acad. Sci. **443**, 171-188. (10.1111/j.1749-6632.1985.tb27072.x) 3860070

[18] Petschenka G, Bramer C, Pankoke H, Dobler S. 2011 Evidence for a deterrent effect of cardenolides on *Nephila* spiders. Basic Appl. Ecol. **12**, 260-267. (10.1016/j.baae.2010.12.005)

[19] Petschenka G et al. 2022 Sequestration of defenses against predators drives specialized host plant associations in preadapted milkweed bugs (Heteroptera: Lygaeinae). Am. Nat. **199**, E211-E228. (10.1086/719196) 35580225

[20] Schoner W, Scheiner-Bobis G. 2007 Endogenous and exogenous cardiac glycosides and their mechanisms of action. Am. J. Cardiovasc. Drugs **7**, 173-189. (10.2165/00129784-200707030-00004) 17610345

[21] Yang L, Ravikanthachari N, Mariño-Pérez R, Deshmukh R, Wu M, Rosenstein A, Kunte K, Song H, Andolfatto P. 2019 Predictability in the evolution of Orthopteran cardenolide insensitivity. Phil. Trans. R. Soc. B **374**, 20180246. (10.1098/rstb.2018.0246) 31154978 PMC6560278

[22] Malcolm S, Brower L. 1989 Evolutionary and ecological implications of cardenolide sequestration in the monarch butterfly. Experientia **45**, 284-295. (10.1007/BF01951814)

[23] Brower L, Moffitt C. 1974 Palatability dynamics of cardenolides in the monarch butterfly. Nature **249**, 280-283. (10.1038/249280b0) 4833249

[24] Brower LP, Van Brower J, Corvino JM. 1967 Plant poisons in a terrestrial food chain. Proc. Natl Acad. Sci. USA **57**, 893-898. (10.1073/pnas.57.4.893) 5231352 PMC224631

[25] Brower LP. 1969 Ecological chemistry. Sci. Am. **220**, 22-29. (10.1038/scientificamerican0269-22) 5767170

[26] Fink LS, Brower LP, Waide RB, Spitzer PR. 1983 Overwintering monarch butterflies as food for insectivorous birds in Mexico. Biotropica **15**, 151-153. (10.2307/2387962)

[27] Brower LP, Horner BE, Marty MA, Moffitt CM, Villa-R B. 1985 Mice (*Peromyscus maniculatus*, *P. spicilegus*, and *Microtus mexicanus*) as predators of overwintering monarch butterflies (*Danaus plexippus*) in Mexico. Biotropica, 89-99. (10.2307/2388500)

[28] Rothschild M, Kellett D. 1972 Reactions of various predators to insects storing heart poisons (cardiac glycosides) in their tissues. J. Entomol. Ser. Gen. Entomol. **46**, 103-110.

[29] Brower LP, Nelson CJ, Seiber J, Fink L, Bond C. 1988 Exaptation as an alternative to coevolution in the cardenolide-based chemical defense of monarch butterflies (*Danaus plexippus* L.) against avian predators. In Chemical mediation of coevolution (ed. KC Spencer), pp. 447-475. New York, NY: Academic Press.

[30] Glendinning JI, Brower LP. 1990 Feeding and breeding responses of five mice species to overwintering aggregations of the monarch butterfly. J. Anim. Ecol. **59**, 1091-1112. (10.2307/5034)

[31] Groen S, Whiteman N. 2021 Convergent evolution of cardiac-glycoside resistance in predators and parasites of milkweed herbivores. Curr. Biol. **31**, R1465-R1466. (10.1016/j.cub.2021.10.025) 34813747 PMC8892682

[32] Brower L, Seiber J, Nelson C, Lynch S, Hoggard M, Cohen J. 1984 Plant-determined variation in cardenolide content and thin-layer chromatography profiles of monarch butterflies, *Danaus plexippus* reared on milkweed plants in California. J. Chem. Ecol. **10**, 1823-1857. (10.1007/BF00987364) 24318436

[33] Black DW. 1976 Studies on cardiac glycoside storage in moths. Miami, FL: University of Miami.

[34] Nishio S. 1980 The fates and adaptive significance of cardenolides sequestered by larvae of *Danaus plexippus* (L.) and *Cycnia inopinatus* (Hy. Edwards). *Diss. Abstr. Int. B Sci. Eng.* **4110**, 3681. See https://eurekamag.com/research/021/926/021926985.php.

[35] Cohen JA, Brower LP. 1982 Oviposition and larval success of wild monarch butterflies (Lepidoptera: Danaidae) in relation to host plant size and cardenolide concentration. J. Kans. Entomol. Soc. **55**, 343-348.

[36] Petschenka G, Offe JK, Dobler S. 2012 Physiological screening for target site insensitivity and localization of Na^+^/K^+^-ATPase in cardenolide-adapted Lepidoptera. J. Insect Physiol. **58**, 607-612. (10.1016/j.jinsphys.2011.12.012) 22343317

[37] Brower LP, McEvoy PB, Williamson KL, Flannery MA. 1972 Variation in cardiac glycoside content of monarch butterflies from natural populations in eastern North America. Science **177**, 426-429. (10.1126/science.177.4047.426) 5043141

[38] Roeske C, Seiber J, Brower L, Moffitt C. 1976 Milkweed cardenolides and their comparative processing by monarch butterflies (*Danaus plexippus* L.). In Biochemical interaction between plants and insects (ed. SJ Segal), pp. 93-167. Berlin, Germany: Springer.

[39] Dobler S, Rowell-Rahier M. 1994 Production of cardenolides versus sequestration of pyrrolizidine alkaloids in larvae of *Oreina* species (Coleoptera, Chrysomelidae). J. Chem. Ecol. **20**, 555-568. (10.1007/BF02059597) 24242111

[40] Van Oycke S, Braekman JC, Daloze D, Pasteels J. 1987 Cardenolide biosynthesis in chrysomelid beetles. Experientia **43**, 460-462. (10.1007/BF01940455)

[41] Fujii Y, Shimada K, Niizaki Y, Nambara T. 1975 Cardenobufotoxin: novel conjugated cardenolide from Japanese toad. Tetrahedron Lett. **16**, 3017-3020. (10.1016/S0040-4039(00)75061-1)

[42] Kingdon J, Agwanda B, Kinnaird M, O'Brien T, Holland C, Gheysens T, Boulet-Audet M, Vollrath F. 2012 A poisonous surprise under the coat of the African crested rat. Proc. R. Soc. B **279**, 675-680. (10.1098/rspb.2011.1169) PMC3248729 21813554

[43] Rodríguez C, Rollins-Smith L, Ibáñez R, Durant-Archibold AA, Gutiérrez M. 2017 Toxins and pharmacologically active compounds from species of the family Bufonidae (Amphibia, Anura). J. Ethnopharmacol. **198**, 235-254. (10.1016/j.jep.2016.12.021) 28034659

[44] Gao H, Zehl M, Leitner A, Wu X, Wang Z, Kopp B. 2010 Comparison of toad venoms from different *Bufo* species by HPLC and LC-DAD-MS/MS. J. Ethnopharmacol. **131**, 368-376. (10.1016/j.jep.2010.07.017) 20637273

[45] Qi J, Zulfiker AHM, Li C, Good D, Wei MQ. 2018 The development of toad toxins as potential therapeutic agents. Toxins **10**, 336. (10.3390/toxins10080336) 30127299 PMC6115759

[46] Verpoorte R, Svendsen AB. 1980 Chemical constituents of Vietnamese toad venom, collected from *Bufo melanostictus* Schneider. Part II. The bufadienolides. J. Nat. Prod. **43**, 347-352. (10.1021/np50009a005) 542013

[47] Steyn PS, van Heerden FR. 1998 Bufadienolides of plant and animal origin. Nat. Prod. Rep. **15**, 397-413. (10.1039/a815397y) 9736996

[48] Córdova WHP et al. 2016 Bufadienolides from parotoid gland secretions of Cuban toad *Peltophryne fustiger* (Bufonidae): inhibition of human kidney Na^+^/K^+^-ATPase activity. Toxicon **110**, 27-34. (10.1016/j.toxicon.2015.11.015) 26615828

[49] Barnhart K, Forman ME, Umile TP, Kueneman J, McKenzie V, Salinas I, Minbiole KP, Woodhams DC. 2017 Identification of bufadienolides from the boreal toad, *Anaxyrus boreas*, active against a fungal pathogen. Microb. Ecol. **74**, 990-1000. (10.1007/s00248-017-0997-8) 28631214

[50] Eisner T, Goetz MA, Hill DE, Smedley SR, Meinwald J. 1997 Firefly ‘femmes fatales’ acquire defensive steroids (lucibufagins) from their firefly prey. Proc. Natl Acad. Sci. USA **94**, 9723-9728. (10.1073/pnas.94.18.9723) 9275191 PMC23257

[51] González A, Schroeder FC, Attygalle AB, Svatoš A, Meinwald J, Eisner T. 1999 Metabolic transformations of acquired lucibufagins by firefly ‘femmes fatales’. Chemoecology **9**, 105-112. (10.1007/s000490050040)

[52] Lloyd JE. 1965 Aggressive mimicry in *Photuris*: firefly femmes fatales. Science **149**, 653-654. (10.1126/science.149.3684.653) 17747574

[53] Lewis SM, Cratsley CK. 2008 Flash signal evolution, mate choice, and predation in fireflies. Annu. Rev. Entomol. **53**, 293-321. (10.1146/annurev.ento.53.103106.093346) 17877452

[54] Yoshida T et al. 2020 Dramatic dietary shift maintains sequestered toxins in chemically defended snakes. Proc. Natl Acad. Sci. USA **117**, 5964-5969. (10.1073/pnas.1919065117) 32094167 PMC7084117

[55] Duffey SS, Scudder GGE. 1972 Cardiac glycosides in North American Asclepiadaceae, a basis for unpalatability in brightly coloured Hemiptera and Coleoptera. J. Insect Physiol. **18**, 63-78. (10.1016/0022-1910(72)90065-0)

[56] Dobler S, Daloze D, Pasteels JM. 1998 Sequestration of plant compounds in a leaf beetle's defensive secretion: cardenolides in *Chrysochus*. Chemoecology **8**, 111-118. (10.1007/s000490050015)

[57] Isman MB, Duffey SS, Scudder GGE. 1977 Cardenolide content of some leaf- and stem-feeding insects on temperate North American milkweeds (*Asclepias* spp.). Can. J. Zool. **55**, 1024-1028. (10.1139/z77-130)

[58] Rothschild M, von Euw J, Reichstein T. 1970 Cardiac glycosides in the oleander aphid, *Aphis nerii*. J. Insect Physiol. **16**, 1141-1145. (10.1016/0022-1910(70)90203-9) 5469742

[59] Duffey SS, Blum MS, Isman MB, Scudder GGE. 1978 Cardiac glycosides: a physical system for their sequestration by the milkweed bug. J. Insect Physiol. **24**, 639-645. (10.1016/0022-1910(78)90127-0)

[60] Euw JV, Fishelson L, Parsons JA, Reichstein T, Rothschild M. 1967 Cardenolides (heart poisons) in a grasshopper feeding on milkweeds. Nature **214**, 35-39. (10.1038/214035a0) 6040609

[61] Poulton EB. 1890 The colours of animals: their meaning and use, especially considered in the case of insects. London, UK: Kegan Paul, Trench & Trubner.

[62] Begon M, Townsend CR. 2020 Ecology: from individuals to ecosystems. New York, NY: John Wiley & Sons.

[63] Mohammadi S, Gompert Z, Gonzalez J, Takeuchi H, Mori A, Savitzky AH. 2016 Toxin-resistant isoforms of Na^+^/K^+^-ATPase in snakes do not closely track dietary specialization on toads. Proc. R. Soc. B **283**, 20162111. (10.1098/rspb.2016.2111) PMC5124105 27852804

[64] Edgren RA, Edgren MK. 1955 Experiments on bluffing and death-feigning in the hognose snake *Heterodon platyrhinos*. Copeia **1955**, 2-4. (10.2307/1439444)

[65] Platt DR. 1967 Natural history of the eastern and the western hognose snakes *Heterodon platyrhinos* and *Heterodon nasicus*. Univ. Kans. Pub. Mus. Nat. Hist. **18**, 253-420.

[66] Cooper WE, Secor S. 2007 Strong response to anuran chemical cues by an extreme dietary specialist, the eastern hog-nosed snake (*Heterodon platirhinos*). Can. J. Zool. **85**, 619-625. (10.1139/Z07-041)

[67] Beane J, Messenger K, Stephan D. 2011 Natural history notes: *Heterodon simus* diet. Herp Rev. **42**, 292.

[68] Durso AM, Mullin SJ. 2017 Ontogenetic shifts in the diet of plains hog-nosed snakes (*Heterodon nasicus*) revealed by stable isotope analysis. Zoology **120**, 83-91. (10.1016/j.zool.2016.07.004) 27692795

[69] Emerson Y, Apolonio JB. 2018 Speckle-bellied keelback *Rhabdophis chrysargos* predation on Philippine toad on Palawan Island, Philippines. *Southeast Asia Vertebrate Records*. See https://www.ecologyasia.com/pdf/2018/seavr2018-011(p028-029).pdf.

[70] Mohammadi S, Hill JG. 2012 Dietary and behavioral notes on the red-necked keelback (*Rhabdophis subminiatus*) from Northeast Thailand. Trop. Nat. Hist. **12**, 123-125.

[71] Akani G, Luiselli L, Tooze Z, Angelici F, Corti C, Zuffi M. 2001 The ecological distribution of *Causus* Wagler 1830 (Viperidae) in Nigeria, with special reference to *C. resimus* (Peters 1862) and *C. lichtensteini* (Jan 1859), two species rarely recorded from this country. Trop. Zool. **14**, 185-195. (10.1080/03946975.2001.10531151)

[72] Loveridge A. 1925 Notes on East African Batrachians, collected 1920–1923, with the description of four new species, pp. 763-791. New York, NY: Wiley Online Library.

[73] Arnold SJ, Wassersug RJ. 1978 Differential predation on metamorphic anurans by garter snakes (*Thamnophis*): social behavior as a possible defense. Ecology **59**, 1014-1022. (10.2307/1938553)

[74] Clark Jr DR. 1974 The western ribbon snake (*Thamnophis proximus*): ecology of a Texas population. Herpetologica **30**, 372-379.

[75] Ernst CH, Ernst EM. 2003 Snakes of the United States and Canada. Washington, DC: Smithsonian Books.

[76] Lavilla E, Scrocchi G, Terán E. 1979 Sobre algunos aspectos del comportamiento en cautiverio de Xenodon merremii (Wagler)(Ophidia: Colubridae). Acta Zool. Lilloana **35**, 287-293.

[77] Beebe W. 1946 Field notes on the snakes of Kartabo. Br. Guiana Caripito Venezuela Zool. **31**, 11-52.

[78] Sidorovich VE, Pikulik MM. 1997 Toads *Bufo* spp. in the diets of mustelid predators in Belarus. Acta Theriol. (Warsz.) **42**, 105-108. (10.4098/AT.arch.97-12)

[79] Cintra R. 1988 *Bufo marinus* (marine toad) predation. Herpetol. Rev. **19**, 82.

[80] Hanson JA, Vial JL. 1956 Defensive behavior and effects of toxins in *Bufo alvarius*. Herpetologica **12**, 141-149.

[81] Brodie Jr ED, Formanowicz Jr DR, Brodie III E. 1978 The development of noxiousness of *Bufo americanus* tadpoles to aquatic insect predators. Herpetologica **34**, 302-306.

[82] Kulkarni M, Adhikari O, Ogale H. 2020 Attack on adult Asian black-spotted toad *Duttaphrynus melanostictus* (Schneider, 1799) by a terrestrial carabid *Epomis* larva. J. Bombay Nat. Hist. Soc. **117**. (10.17087/jbnhs/2020/v117/150540)

[83] Elron E, Shlagman A, Gasith A. 2007 First detailed report of predation on anuran metamorphs by terrestrial beetle larvae. Herpetol. Rev. **38**, 30-32.

[84] Escoriza D, Mestre L, Pascual G, Buse J. 2017 First case of attack of an adult *Bufo spinosus* Daudin, 1803 by a carabid beetle larva of *Epomis circumscriptus* (Duftschmid, 1812). Bol. Asoc. Herpetológica Esp. **28**, 51-52.

[85] Wizen G, Gasith A. 2011 Predation of amphibians by carabid beetles of the genus *Epomis* found in the central coastal plain of Israel. ZooKeys **100**, 181-191. (10.3897/zookeys.100.1526) PMC3131015 21738411

[86] Urquhart FA. 1960 The monarch butterfly. Toronto, Canada: University of Toronto Press.

[87] Smithers C. 1973 A note on length of adult life of some Australian butterflies. Aust. Entomol. **1**, 62.

[88] Zalucki M, Kitching R. 1982 Dynamics of oviposition in *Danaus plexippus* (Insecta: Lepidoptera) on milkweed, *Asclepias* spp. J. Zool. **198**, 103-116. (10.1111/j.1469-7998.1982.tb02063.x)

[89] De Anda A, Oberhauser KS. 2015 Invertebrate natural enemies and stage-specific mortality rates of monarch eggs and larvae. In Monarchs in a changing world: biology and conservation of an iconic butterfly (eds KS Oberhauser, KR Nail, S Altizer), pp. 60-70. Ithaca, NY: Cornell University Press.

[90] Oberhauser KS, Nail KR, Altizer S. 2015 Monarchs in a changing world: biology and conservation of an iconic butterfly. Ithaca, NY: Cornell University Press.

[91] Hermann SL, Blackledge C, Haan NL, Myers AT, Landis DA. 2019 Predators of monarch butterfly eggs and neonate larvae are more diverse than previously recognised. Sci. Rep. **9**, 1-9. (10.1038/s41598-018-37186-2) 31586127 PMC6778129

[92] Calvert WH, Hedrick LE, Brower LP. 1979 Mortality of the monarch butterfly (*Danaus plexippus* L.): avian predation at five overwintering sites in Mexico. Science **204**, 847-851. (10.1126/science.204.4395.847) 17730529

[93] Rayor LS. 2004 Effects of monarch larval host plant chemistry and body size on *Polistes* wasp predation. In Monarch butterfly biology and conserv. (eds K Oberhauser, M Solensky), pp. 39-46. Ithaca, NY: Cornell University Press.

[94] Day JC. 2011 Parasites, predators and defence of fireflies and glow-worms. Lampyrid **1**, 70-102.

[95] Hamilton Jr WJ. 1933 The insect food of the big brown bat. J. Mammal. **14**, 155-156. (10.1093/jmammal/14.2.155)

[96] Sexton OJ, Hoger C, Ortleb E. 1966 *Anolis carolinensis*: effects of feeding on reaction to aposematic prey. Science **153**, 1140. (10.1126/science.153.3740.1140) 17737596

[97] Pagani-Núñez E, Barnett C, Gu H, Goodale E. 2016 The need for new categorizations of dietary specialism incorporating spatio-temporal variability of individual diet specialization. J. Zool. **300**, 1-7. (10.1111/jzo.12364)

[98] Pyke GH, Pulliam HR, Charnov EL. 1977 Optimal foraging: a selective review of theory and tests. Q. Rev. Biol. **52**, 137-154. (10.1086/409852)

[99] Banks B, Beebee T. 1987 Spawn predation and larval growth inhibition as mechanisms for niche separation in anurans. Oecologia **72**, 569-573. (10.1007/BF00378984) 28312520

[100] Svádová K, Exnerova A, Štys P, Landova E, Valenta J, Fučíková A, Socha R. 2009 Role of different colours of aposematic insects in learning, memory and generalization of naïve bird predators. Anim. Behav. **77**, 327-336. (10.1016/j.anbehav.2008.09.034)

[101] Halpin CG, Penacchio O, Lovell PG, Cuthill I, Harris J, Skelhorn J, Rowe C. 2020 Pattern contrast influences wariness in naïve predators towards aposematic patterns. Sci. Rep. **10**, 1-8. (10.1038/s41598-020-65754-y) 32514003 PMC7280217

[102] Brower LP, Calvert WH. 1985 Foraging dynamics of bird predators on overwintering monarch butterflies in Mexico. Evolution **39**, 852-868. (10.1111/j.1558-5646.1985.tb00427.x) 28561370

[103] Visalberghi E, Addessi E. 2000 Seeing group members eating a familiar food enhances the acceptance of novel foods in capuchin monkeys. Anim. Behav. **60**, 69-76. (10.1006/anbe.2000.1425) 10924205

[104] Hämäläinen L, Hoppitt W, Rowland HM, Mappes J, Fulford AJ, Sosa S, Thorogood R. 2021 Social transmission in the wild can reduce predation pressure on novel prey signals. Nat. Commun. **12**, 1-11. (10.1038/s41467-021-24154-0) 34172738 PMC8233390

[105] Donato D, Potts R. 2004 Culturally transmitted predation and consumption techniques by Torresian crows *Corvus orru* on cane toads *Bufo marinus*. Aust. Field Ornithol. **21**, 125-126.

[106] Franks VR, Ewen JG, McCready M, Thorogood R. 2020 Foraging behaviour alters with social environment in a juvenile songbird. Proc. R. Soc. B **287**, 20201878. (10.1098/rspb.2020.1878) PMC7739504 33234077

[107] Page RA, Ryan MJ. 2006 Social transmission of novel foraging behavior in bats: frog calls and their referents. Curr. Biol. **16**, 1201-1205. (10.1016/j.cub.2006.04.038) 16782010

[108] Rowe C, Halpin C. 2013 Why are warning displays multimodal? Behav. Ecol. Sociobiol. **67**, 1425-1439. (10.1007/s00265-013-1515-8)

[109] Brooke M de L. 2019 Is eliciting disgust responses from its predators beneficial for toxic prey? Anim. Behav. **155**, 225-227. (10.1016/j.anbehav.2019.07.007)

[110] Rafter JL, Agrawal AA, Preisser EL. 2013 Chinese mantids gut toxic monarch caterpillars: avoidance of prey defence? Ecol. Entomol. **38**, 76-82. (10.1111/j.1365-2311.2012.01408.x)

[111] Mebs D, Wunder C, Pogoda W, Toennes SW. 2017 Feeding on toxic prey: the praying mantis (Mantodea) as predator of poisonous butterfly and moth (Lepidoptera) caterpillars. Toxicon **131**, 16-19. (10.1016/j.toxicon.2017.03.010) 28300580

[112] Leong K, Frey D, Nagano C. 1990 Wasp predation on overwintering monarch butterflies (Lepidoptera: Danaidae) in central California. Pan-Pac. Entomol. **66**, 326-328.

[113] Almeida D, Rodolfo N, Sayer CD, Copp GH. 2013 Seasonal use of ponds as foraging habitat by Eurasian otter with description of an alternative handling technique for common toad predation. J. Vertebr. Biol. **62**, 214-221.

[114] Slater F. 2002 Progressive skinning of toads (*Bufo bufo*) by the Eurasian otter (*Lutra lutra*). IUCN Otter Spec. Group Bull. **19**, 25-29.

[115] Bringsøe H, Suthanthangjai M, Suthanthangjai W, Nimnuam K. 2020 Eviscerated alive: novel and macabre feeding strategy in *Oligodon fasciolatus* (Günther, 1864) eating organs of *Duttaphrynus melanostictus* (Schneider, 1799) in Thailand. Herpetozoa **33**, 157. (10.3897/herpetozoa.33.e57096)

[116] Evans AM, Choiniere JN, Alexander GJ. 2019 The cutting-edge morphology of the mole snake's dental apparatus. PeerJ **7**, e6943. (10.7717/peerj.6943) 31211009 PMC6557247

[117] Morales J, Ruiz-Olmo J, Lizana M, Gutiérrez J. 2016 Skinning toads is innate behaviour in otter (*Lutra lutra*) cubs. Ethol. Ecol. Evol. **28**, 414-426. (10.1080/03949370.2015.1076525)

[118] Groves JD. 1980 Mass predation on a population of the American toad, *Bufo americanus*. Am. Midl. Nat. **103**, 202-203. (10.2307/2425057)

[119] Thorogood R, Kokko H, Mappes J. 2018 Social transmission of avoidance among predators facilitates the spread of novel prey. Nat. Ecol. Evol. **2**, 254-261. (10.1038/s41559-017-0418-x) 29255302

[120] Fink LS, Brower LP. 1981 Birds can overcome the cardenolide defence of monarch butterflies in Mexico. Nature **291**, 67-70. (10.1038/291067a0)

[121] Malcolm SB. 1991 Cardenolide-mediated interactions between plants and herbivores. In Herbivores: their interactions with secondary plant metabolites, vol. 1 (eds GA Rosenthal, MR Berenbaum), pp. 251-296. San Diego, CA: Academic Press.

[122] Fukuda M, Mori A. 2021 Does an Asian natricine snake, *Rhabdophis tigrinus*, have chemical preference for a skin toxin of toads? Curr. Herpetol. **40**, 1-9. (10.5358/hsj.40.1)

[123] Liu Z, Liu G, Hailer F, Orozco-terWengel P, Tan X, Tian J, Yan Z, Zhang B, Li M. 2016 Dietary specialization drives multiple independent losses and gains in the bitter taste gene repertoire of Laurasiatherian Mammals. Front. Zool. **13**, 28. (10.1186/s12983-016-0161-1) 27366197 PMC4928315

[124] Shan L, Wu Q, Wang L, Zhang L, Wei F. 2018 Lineage-specific evolution of bitter taste receptor genes in the giant and red pandas implies dietary adaptation. Integr. Zool. **13**, 152-159. (10.1111/1749-4877.12291) 29168616 PMC5873442

[125] Mohammadi S, Yang L, Herrera-Álvarez S, del Pilar Rodríguez-Ordoñez M, Zhang K, Storz JF, Dobler S, Crawford AJ, Andolfatto P. 2022 Constraints on the evolution of toxin-resistant Na,K-ATPases have limited dependence on sequence divergence. *bioRxiv*, 2021.11.29.470343. (10.1101/2021.11.29.470343)

[126] Ujvari B et al. 2015 Widespread convergence in toxin resistance by predictable molecular evolution. Proc. Natl Acad. Sci. USA **112**, 11 911-11 916. (10.1073/pnas.1511706112) PMC4586833 26372961

[127] Moore DJ, Halliday DC, Rowell DM, Robinson AJ, Keogh JS. 2009 Positive Darwinian selection results in resistance to cardioactive toxins in true toads (Anura: Bufonidae). Biol. Lett. **5**, 513-516. (10.1098/rsbl.2009.0281) 19465576 PMC2781935

[128] Mohammadi S et al. 2021 Concerted evolution reveals co-adapted amino acid substitutions in frogs that prey on toxic toads. Curr. Biol. **31**, 2530-2538.e10. (10.1016/j.cub.2021.03.089) 33887183 PMC8281379

[129] Marshall BM, Casewell NR, Vences M, Glaw F, Andreone F, Rakotoarison A, Zancolli G, Woog F, Wüster W. 2018 Widespread vulnerability of Malagasy predators to the toxins of an introduced toad. Curr. Biol. **28**, R654-R655. (10.1016/j.cub.2018.04.024) 29870701

[130] Price EM, Rice DA, Lingrel JB. 1990 Structure-function studies of Na, K-ATPase. Site-directed mutagenesis of the border residues from the H1-H2 extracellular domain of the alpha subunit. J. Biol. Chem. **265**, 6638-6641. (10.1016/S0021-9258(19)39197-5) 2157705

[131] Carpenter GH. 1942 Observations and experiments in Africa by the late CFM Swynnerton on wild birds eating butterflies and the preference shown. pp. 10-46. Oxford, UK: Oxford University Press.

[132] Thompson JN. 2005 The geographic mosaic of coevolution. Chicago, IL: University of Chicago Press.

[133] Zhen Y, Aardema ML, Medina EM, Schumer M, Andolfatto P. 2012 Parallel molecular evolution in an herbivore community. Science **337**, 1634-1637. (10.1126/science.1226630) 23019645 PMC3770729

[134] Agrawal AA, Böröczky K, Haribal M, Hastings AP, White RA, Jiang R-W, Duplais C. 2021 Cardenolides, toxicity, and the costs of sequestration in the coevolutionary interaction between monarchs and milkweeds. Proc. Natl Acad. Sci. USA **118**, e2024463118. (10.1073/pnas.2024463118) 33850021 PMC8072370

[135] Groen SC, LaPlante ER, Alexandre NM, Agrawal AA, Dobler S, Whiteman NK. 2017 Multidrug transporters and organic anion transporting polypeptides protect insects against the toxic effects of cardenolides. Insect Biochem. Mol. Biol. **81**, 51-61. (10.1016/j.ibmb.2016.12.008) 28011348 PMC5428987

[136] Marzo A, Ghirardi P. 1977 Biliary and urinary excretion of five cardiac glycosides and its correlation with their physical and chemical properties. Naunyn. Schmiedebergs Arch. Pharmacol. **298**, 51-56. (10.1007/BF00510986) 882147

[137] Brower LP, Seiber JN, Nelson CJ, Lynch SP, Tuskes PM. 1982 Plant-determined variation in the cardenolide content, thin-layer chromatography profiles, and emetic potency of monarch butterflies, *Danaus plexippus* reared on the milkweed, *Asclepias eriocarpa* in California. J. Chem. Ecol. **8**, 579-633. (10.1007/BF00989631) 24415043

[138] Nelson CJ, Seiber JN, Brower LP. 1981 Seasonal and intraplant variation of cardenolide content in the California milkweed, *Asclepias eriocarpa*, and implications for plant defense. J. Chem. Ecol. **7**, 981-1010. (10.1007/BF00987622) 24420825

[139] Petschenka G, Dobler S. 2009 Target-site sensitivity in a specialized herbivore towards major toxic compounds of its host plant: the Na^+^ K^+^-ATPase of the oleander hawk moth (Daphnisnerii) is highly susceptible to cardenolides. Chemoecology **19**, 235. (10.1007/s00049-009-0025-7)

[140] Karageorgi M et al. 2019 Genome editing retraces the evolution of toxin resistance in the monarch butterfly. Nature **574**, 409-412. (10.1038/s41586-019-1610-8) 31578524 PMC7039281

[141] Dobler S, Petschenka G, Wagschal V, Flacht L. 2015 Convergent adaptive evolution – how insects master the challenge of cardiac glycoside-containing host plants. Entomol. Exp. Appl. **157**, 30-39. (10.1111/eea.12340)

[142] Scudder GGE, Meredith J. 1982 The permeability of the midgut of three insects to cardiac glycosides. J. Insect Physiol. **28**, 689-694. (10.1016/0022-1910(82)90147-0)

[143] Haiser HJ, Gootenberg DB, Chatman K, Sirasani G, Balskus EP, Turnbaugh PJ. 2013 Predicting and manipulating cardiac drug inactivation by the human gut bacterium *Eggerthella lenta*. Science **341**, 295-298. (10.1126/science.1235872) 23869020 PMC3736355

[144] Cavet ME, West M, Simmons NL. 1996 Transport and epithelial secretion of the cardiac glycoside, digoxin, by human intestinal epithelial (CaCo-2) cells. Br. J. Pharmacol. **118**, 1389-1396. (10.1111/j.1476-5381.1996.tb15550.x) 8832062 PMC1909679

[145] Mayer U, Wagenaar E, Beijnen JH, Smit JW, Meijer DK, van Asperen J, Borst P, Schinkel AH. 1996 Substantial excretion of digoxin via the intestinal mucosa and prevention of long-term digoxin accumulation in the brain by the mdrla P-glycoprotein. Br. J. Pharmacol. **119**, 1038-1044. (10.1111/j.1476-5381.1996.tb15775.x) 8922756 PMC1915939

[146] Schinkel AH, Wagenaar E, van Deemter L, Mol C, Borst P. 1995 Absence of the mdr1a P-glycoprotein in mice affects tissue distribution and pharmacokinetics of dexamethasone, digoxin, and cyclosporin A. J. Clin. Invest. **96**, 1698-1705. (10.1172/JCI118214) 7560060 PMC185805

[147] Abderemane-Ali F et al. 2021 Evidence that toxin resistance in poison birds and frogs is not rooted in sodium channel mutations and may rely on ‘toxin sponge’ proteins. J. Gen. Physiol. **153**, e20212872. (10.1085/jgp.202112872) PMC8348241 34351379

[148] Antolovic R, Bauer N, Mohadjerani M, Kost H, Neu H, Kirch U, Grünbaum E-G, Schoner W. 2000 Endogenous ouabain and its binding globulin: effects of physical exercise and study on the globulin's tissue distribution. Hypertens. Res. **23**, S93-S98. (10.1291/hypres.23.Supplement_S93) 11016826

[149] Antolovic R, Kost H, Mohadjerani M, Linder D, Linder M, Schoner W. 1998 A specific binding protein for cardiac glycosides exists in bovine serum. J. Biol. Chem. **273**, 16 259-16 264. (10.1074/jbc.273.26.16259) 9632685

[150] Schoner W et al. 2003 Ouabain as a mammalian hormone. Ann. N. Y. Acad. Sci. **986**, 678-684. (10.1111/j.1749-6632.2003.tb07282.x) 12763918

[151] Mohammadi S, McCoy K, Hutchinson D, Gauthier D, Savitzky A. 2013 Independently evolved toad-eating snakes exhibit sexually dimorphic enlargement of adrenal glands. J. Zool. **290**, 237-245. (10.1111/jzo.12038)

[152] Mohammadi S, French SS, Neuman-Lee LA, Durham SL, Kojima Y, Mori A, Brodie Jr ED, Savitzky AH. 2017 Corticosteroid responses of snakes to toxins from toads (bufadienolides) and plants (cardenolides) reflect differences in dietary specializations. Gen. Comp. Endocrinol. **247**, 16-25. (10.1016/j.ygcen.2017.03.015) 28347742

[153] Summa V, Mordasini D, Roger F, Bens M, Martin P-Y, Vandewalle A, Verrey F, Féraille E. 2001 Short term effect of aldosterone on Na, K-ATPase cell surface expression in kidney collecting duct cells. J. Biol. Chem. **276**, 47 087-47 093. (10.1074/jbc.M107165200) 11598118

[154] Feraille E, Dizin E. 2016 Coordinated control of ENaC and Na^+^, K^+^-ATPase in renal collecting duct. J. Am. Soc. Nephrol. **27**, 2554-2563. (10.1681/ASN.2016020124) 27188842 PMC5004664

[155] Ikeda U, Hyman R, Smith TW, Medford RM. 1991 Aldosterone-mediated regulation of Na^+^, K^+^-ATPase gene expression in adult and neonatal rat cardiocytes. J. Biol. Chem. **266**, 12 058-12 066. (10.1016/S0021-9258(18)99065-4) 1646819

[156] Oguchi A, Ikeda U, Kanbe T, Tsuruya Y, Yamamoto K, Kawakami K, Medford RM, Shimada K. 1993 Regulation of Na-K-ATPase gene expression by aldosterone in vascular smooth muscle cells. Am. J. Physiol.-Heart Circ. Physiol. **265**, H1167-H1172. (10.1152/ajpheart.1993.265.4.H1167) 8238401

[157] Mohammadi S, Savitzky AH, Lohr J, Dobler S. 2017 Toad toxin-resistant snake (*Thamnophis elegans*) expresses high levels of mutant Na^+^/K^+^-ATPase mRNA in cardiac muscle. Gene **614**, 21-25. (10.1016/j.gene.2017.02.028) 28249773

[158] Kumar K, Jaiswal SK, Dhoke GV, Srivastava GN, Sharma AK, Sharma VK. 2018 Mechanistic and structural insight into promiscuity based metabolism of cardiac drug digoxin by gut microbial enzyme. J. Cell. Biochem. **119**, 5287-5296. (10.1002/jcb.26638) 29274283

[159] Koppel N, Bisanz JE, Pandelia M-E, Turnbaugh PJ, Balskus EP. 2018 Discovery and characterization of a prevalent human gut bacterial enzyme sufficient for the inactivation of a family of plant toxins. eLife **7**, e33953. (10.7554/eLife.33953) 29761785 PMC5953540

[160] Jackson KA, McCord JS, White JA. 2017 A window of opportunity: subdominant predators can use suboptimal prey. Ecol. Evol. **7**, 5269-5275. (10.1002/ece3.3139) 28770065 PMC5528202

[161] Brodie ED. 1977 Hedgehogs use toad venom in their own defence. Nature **268**, 627-628. (10.1038/268627a0)

[162] Mori A, Burghardt GM. 2000 Does prey matter? Geographic variation in antipredator responses of hatchlings of a Japanese natricine snake (*Rhabdophis tigrinus*). J. Comp. Psychol. **114**, 408. (10.1037/0735-7036.114.4.408) 11149545

[163] Kojima Y, Mori A. 2015 Active foraging for toxic prey during gestation in a snake with maternal provisioning of sequestered chemical defences. Proc. R. Soc. B **282**, 20142137. (10.1098/rspb.2014.2137) PMC4262175 25392472

[164] Hutchinson DA, Savitzky AH, Mori A, Meinwald J, Schroeder FC. 2008 Maternal provisioning of sequestered defensive steroids by the Asian snake *Rhabdophis tigrinus*. Chemoecology **18**, 181-190. (10.1007/s00049-008-0404-5)

[165] Douglas TE, Beskid SG, Gernand CE, Nirtaut BE, Tamsil KE, Fitch RW, Tarvin RD. 2022 Trade-offs between cost of ingestion and rate of intake drive defensive toxin use. Biol. Lett. **18**, 20210579. (10.1098/rsbl.2021.0579) 35135316 PMC8826133

[166] Andrade-Zuñiga EM, Morales M, Ariano-Sánchez D. 2018 Toxicity of the feathers of Yellow Grosbeak, *Pheucticus chrysopeplus* (Passeriformes: Cardinalidae), a chemically defended neotropical bird. Rev. Biol. Trop. **66**, 1530-1535. (10.15517/rbt.v66i4.32059)

[167] Mutoh A. 1983 Death-feigning behavior of the Japanese colubrid snake *Rhabdophis tigrinus*. Herpetologica **39**, 78-80.

[168] Gregory PT, Isaac LA, Griffiths RA. 2007 Death feigning by grass snakes (*Natrix natrix*) in response to handling by human ‘predators’. J. Comp. Psychol. Wash. DC 1983 **121**, 123-129. (10.1037/0735-7036.121.2.123) 17516791

[169] Durso AM, Mullin SJ. 2014 Intrinsic and extrinsic factors influence expression of defensive behavior in plains hog-nosed snakes (*Heterodon nasicus*). Ethology **120**, 140-148. (10.1111/eth.12188)

[170] McDonald HS. 1974 Bradycardia during death-feigning of *Heterodon platyrhinos* Latreille (Serpentes). J. Herpetol. **8**, 157-164. (10.2307/1562812)

[171] Savitzky AH, Mori A, Hutchinson DA, Saporito RA, Burghardt GM, Lillywhite HB, Meinwald J. 2012 Sequestered defensive toxins in tetrapod vertebrates: principles, patterns, and prospects for future studies. Chemoecology **22**, 141-158. (10.1007/s00049-012-0112-z) 22904605 PMC3418492

[172] Huffman MA. 2003 Animal self-medication and ethno-medicine: exploration and exploitation of the medicinal properties of plants. Proc. Nutr. Soc. **62**, 371-381. (10.1079/PNS2003257) 14506884

[173] Johnson PT, Calhoun DM, Stokes AN, Susbilla CB, McDevitt-Galles T, Briggs CJ, Hoverman JT, Tkach VV, de Roode JC. 2018 Of poisons and parasites—the defensive role of tetrodotoxin against infections in newts. J. Anim. Ecol. **87**, 1192-1204. (10.1111/1365-2656.12816) 29476541

[174] Lozano GA. 1998 Parasitic stress and self-medication in wild animals. Adv. Study Behav. **27**, 291-318. (10.1016/S0065-3454(08)60367-8)

[175] Cunha Filho GA et al. 2005 Antimicrobial activity of the bufadienolides marinobufagin and telocinobufagin isolated as major components from skin secretion of the toad *Bufo rubescens*. Toxicon **45**, 777-782. (10.1016/j.toxicon.2005.01.017) 15804527

[176] Parker BJ, Barribeau SM, Laughton AM, de Roode JC, Gerardo NM. 2011 Non-immunological defense in an evolutionary framework. Trends Ecol. Evol. **26**, 242-248. (10.1016/j.tree.2011.02.005) 21435735

[177] Rayor LS, Mooney LJ, Renwick JA. 2007 Predatory behavior of *Polistes dominulus* wasps in response to cardenolides and glucosinolates in *Pieris napi* caterpillars. J. Chem. Ecol. **33**, 1177-1185. (10.1007/s10886-007-9283-4) 17453324

[178] Dearing MD. 2013 Temperature-dependent toxicity in mammals with implications for herbivores: a review. J. Comp. Physiol. B **183**, 43-50. (10.1007/s00360-012-0670-y) 22581072

[179] Keplinger ML, Lanier GE, Deichmann WB. 1959 Effects of environmental temperature on the acute toxicity of a number of compounds in rats. Toxicol. Appl. Pharmacol. **1**, 156-161. (10.1016/0041-008X(59)90136-X) 13635685

[180] Schaefer TL, Lingrel JB, Moseley AE, Vorhees CV, Williams MT. 2011 Targeted mutations in the Na, K-ATPase alpha 2 isoform confer ouabain resistance and result in abnormal behavior in mice. Synapse **65**, 520-531. (10.1002/syn.20870) 20936682 PMC3070835

[181] Phillips BL, Brown GP, Shine R. 2003 Assessing the potential impact of cane toads on Australian snakes. Conserv. Biol. **17**, 1738-1747. (10.1111/j.1523-1739.2003.00353.x)

[182] Llewelyn JS, Phillips BL, Shine R. 2009 Sublethal costs associated with the consumption of toxic prey by snakes. Austral Ecol. **34**, 179-184. (10.1111/j.1442-9993.2008.01919.x)

[183] Taverner AM et al. 2019 Adaptive substitutions underlying cardiac glycoside insensitivity in insects exhibit epistasis *in vivo*. eLife **8**, e48224. (10.7554/eLife.48224) 31453806 PMC6733596

[184] Feldman CR, Brodie Jr ED, Brodie III ED, Pfrender ME. 2009 The evolutionary origins of beneficial alleles during the repeated adaptation of garter snakes to deadly prey. Proc. Natl Acad. Sci. USA **106**, 13 415-13 420. (10.1073/pnas.0901224106) PMC2726340 19666534

[185] Reimche JS et al. 2020 The geographic mosaic in parallel: matching patterns of newt tetrodotoxin levels and snake resistance in multiple predator–prey pairs. J. Anim. Ecol. **89**, 1645-1657. (10.1111/1365-2656.13212) 32198924

[186] Brodie ED, Feldman CR, Hanifin CT, Motychak JE, Mulcahy DG, Williams BL. 2005 Parallel arms races between garter snakes and newts involving tetrodotoxin as the phenotypic interface of coevolution. J. Chem. Ecol. **31**, 343-356. (10.1007/s10886-005-1345-x) 15856788

[187] Vermeij GJ. 1993 Evolution and escalation: an ecological history of life. Princeton, NJ: Princeton University Press.

[188] Stephens DW, Krebs JR. 2019 Foraging theory. Princeton, NJ: Princeton University Press.

[189] Grubb JC. 1972 Differential predation by *Gambusia affinis* on the eggs of seven species of anuran amphibians. Am. Midl. Nat. **88**, 102-108. (10.2307/2424491)

[190] Kruse KC, Stone BM. 1984 Largemouth bass (*Micropterus salmoides*) learn to avoid feeding on toad (*Bufo*) tadpoles. Anim. Behav. **32**, 1035-1039. (10.1016/S0003-3472(84)80218-3)

[191] Durso AM, Neuman-Lee LA, Hopkins GR, Brodie Jr ED. 2021 Stable isotope analysis suggests that tetrodotoxin-resistant common gartersnakes (*Thamnophis sirtalis*) rarely feed on newts in the wild. Can. J. Zool. **99**, 331-338. (10.1139/cjz-2020-0215)

[192] Beckmann C, Shine R. 2009 Impact of invasive cane toads on Australian birds. Conserv. Biol. **23**, 1544-1549. (10.1111/j.1523-1739.2009.01261.x) 19508674

[193] Sharom FJ, Holland IB. 2011 ABC transporters, mechanisms and biology: an overview. Essays Biochem. **50**, 1-17. (10.1042/bse0500001) 21967049

[194] Dean M, Annilo T. 2005 Evolution of the ATP-binding cassette (ABC) transporter superfamily in vertebrates. Annu. Rev. Genomics Hum. Genet. **6**, 123-142. (10.1146/annurev.genom.6.080604.162122) 16124856

[195] Ma S et al. 2020 Molecular evolution of the ATP-binding cassette subfamily G member 2 gene subfamily and its paralogs in birds. BMC Evol. Biol. **20**, 85. (10.1186/s12862-020-01654-z) 32664916 PMC7362505

[196] Kowalski P, Baum M, Körten M, Donath A, Dobler S. 2020 ABCB transporters in a leaf beetle respond to sequestered plant toxins. Proc. R. Soc. B **287**, 20201311. (10.1098/rspb.2020.1311) PMC7542790 32873204

[197] Fukase T, Itagaki H, Wakui S, Kano Y, Goris R, Kishida R. 1987 Parasitism of *Pharyngostomum cordatum* Metacercariae (Trematode; Diplostomatidae) in snakes, *Elaphe quadrivirgata* and *Rhabdophis tigrinus* (Reptilia; Colubridae). Jpn. J. Herpetol. **12**, 39-44. (10.5358/hsj1972.12.2_39)

[198] Sato K, Tsuboi T, Torii M, Hirai K, Shiwaku K. 1992 Incidence of the plerocercoids of *Spirometra erinacei* in snakes, *Elaphe quadrivirgata* and *Rhabdophis tigrinus* tigrinus captured in Ehime Prefecture, Japan. Kiseichugaku Zasshi **41**, 340-343. (10.11150/kansenshogakuzasshi1970.66.340)

[199] Mohammadi S, Yang L, Bulbert M, Rowland HM. 2022 Data from: Defence mitigation by predators of chemically defended prey integrated over the predation sequence and across biological levels with a focus on cardiotonic steroids. *Figshare*. (10.6084/m9.figshare.c.6168216) PMC9449480 36133149

